# Impact of Preoperative Obstructive Sleep Apnea on Complications, Healthcare Utilization, and Revision Rates Following Primary Total Knee and Total Hip Arthroplasty: A Systematic Review and Meta-Analysis

**DOI:** 10.7759/cureus.111137

**Published:** 2026-06-19

**Authors:** Neal Naveen, Kenneth Sabacinski, Michael Booth

**Affiliations:** 1 Orthodontics, Ohio State University, Columbus, USA; 2 Orthopaedics, Beth Israel Deaconess Medical Center, Boston, USA; 3 Orthopaedics, Ohio State University Wexner Medical Center, Columbus, USA

**Keywords:** obstructive sleep apnoea, orthopaedics surgery, total hip arthroplasty (tha), total joint arthroplasties, total knee

## Abstract

This systematic review and meta-analysis was conducted in accordance with the Preferred Reporting Items for Systematic Reviews and Meta-Analyses (PRISMA) guidelines. The protocol was registered with PROSPERO (registration number: 420261397468). Obstructive sleep apnea (OSA) affects an estimated 30-40% of patients undergoing total joint arthroplasty (TJA) and is mechanistically linked to systemic inflammation, hypercoagulability, and impaired wound healing. Despite this, OSA has not been comprehensively evaluated as a standalone preoperative predictor of arthroplasty outcomes. This study aimed to quantify the independent effect of preoperative OSA on postoperative complications, healthcare utilization, and implant-related outcomes following primary total knee arthroplasty (TKA) and total hip arthroplasty (THA). A comprehensive search of PubMed, EMBASE, Cochrane Library, and Scopus was conducted from inception to May 1, 2026. Studies comparing postoperative outcomes in patients with and without a preoperative OSA diagnosis undergoing primary TKA or THA were included. Primary outcomes were pulmonary embolism (PE), deep vein thrombosis (DVT), periprosthetic joint infection (PJI), surgical site infection (SSI), and 90-day readmission. Secondary outcomes included length of stay (LOS) and revision rates. Functional outcomes were assessed narratively. Random-effects meta-analysis was performed using the DerSimonian-Laird method; risk of bias was assessed using Risk Of Bias In Non-randomized Studies of Interventions (ROBINS-I). Seventeen studies encompassing approximately 392,000 patients met the inclusion criteria; 15 contributed to at least one pooled analysis. Studies involving spine surgery and shoulder arthroplasty were retained for qualitative synthesis only. OSA diagnosis was independently associated with significantly elevated risk of PE (OR: 1.58, 95% CI: 1.41-1.77; p<0.001; k=7), DVT (OR: 1.45, 95% CI: 1.19-1.76; p<0.001; k=7), PJI (OR: 1.42, 95% CI: 1.24-1.62; p<0.001; k=4), 90-day readmission (OR: 1.57, 95% CI: 1.36-1.82; p<0.001; k=7; sensitivity analysis, excluding CPAP-severity-selection outlier: OR 1.22, 95% CI 1.12-1.33), and revision arthroplasty (OR: 1.26, 95% CI: 1.10-1.45; p=0.001; k=4). SSI data from two TKA/THA studies (OR range 1.23-1.37) were insufficient for formal pooling and are reported narratively. Mean LOS was significantly longer in OSA patients (MD +0.82 days, 95% CI: 0.41-1.23; p<0.001). Preoperative CPAP therapy attenuated thromboembolic but not infectious complication rates. Functional outcome data were insufficient for pooled analysis. Preoperative OSA diagnosis is independently associated with a broad spectrum of complications following primary TKA and THA. These findings support OSA as a target for routine preoperative screening and optimization in arthroplasty candidates, although causal inference is limited by the observational nature of included studies.

## Introduction and background

Total knee arthroplasty (TKA) and total hip arthroplasty (THA) are among the most frequently performed elective orthopaedic procedures worldwide, with demand projected to grow substantially over the coming decades [1]. As arthroplasty volume increases, optimizing preoperative risk stratification has become a priority for orthopaedic surgeons and health systems. Modifiable comorbidities, such as obesity, diabetes mellitus, and depression, have received growing attention as preoperative targets for intervention to reduce postoperative complications.

Obstructive sleep apnea (OSA) is a highly prevalent yet frequently underrecognized comorbidity in the arthroplasty population. The condition affects an estimated 936 million individuals globally and is present in approximately 30-40% of patients undergoing total joint arthroplasty [2]. OSA is characterized by repetitive upper airway obstruction during sleep, producing intermittent hypoxia, sympathetic nervous system hyperactivation, and systemic inflammation. These pathophysiological effects are mechanistically linked to multiple recognized arthroplasty risk factors: enhanced coagulability via increased hematocrit, blood viscosity, and platelet aggregability; impaired neutrophil oxidative burst capacity; disrupted wound healing; and elevated rates of cardiovascular comorbidity [3]. These mechanisms provide a biologically plausible basis for associations between preoperative OSA and thromboembolic, pulmonary, infectious, and implant-related complications.

It is important to distinguish several related but distinct constructs that appear throughout the OSA-arthroplasty literature. A formal OSA diagnosis (typically based on polysomnography-confirmed apnoea-hypopnoea index (AHI) ≥ 15 events/hour, or International Classification of Diseases (ICD) coding in administrative databases) differs from suspected OSA, OSA severity (mild, moderate, severe by AHI), and continuous positive airway pressure (CPAP)-treated OSA. CPAP prescription identifies a subset of patients with clinically significant OSA who are more likely to have moderate-to-severe disease; CPAP use is therefore a marker of both treatment and disease severity, not a simple therapeutic exposure [4]. These distinctions are important for interpreting the heterogeneity in the literature and are addressed in the subgroup analyses below.

Despite the biological plausibility of OSA as a meaningful predictor of arthroplasty complications, the published literature has remained fragmented. Individual studies using large administrative databases have reported elevated rates of specific complications, such as pulmonary embolism (PE), surgical site infection (SSI), and readmission, but have differed in methodology, follow-up, and outcome definitions. A 2024 systematic review by Thomas et al. included seven single-institution studies and reported elevated thromboembolic composite outcomes and pulmonary complication rates in OSA patients undergoing TJA, but was limited in scope and did not evaluate periprosthetic joint infection (PJI), revision rates, or the modifying effect of CPAP therapy [5]. Several large propensity-matched database studies published in 2024-2025 have substantially expanded the available evidence base.

The objective of this systematic review and meta-analysis was to provide a comprehensive, updated synthesis of evidence on the association between preoperative OSA diagnosis and outcomes following primary TKA and THA. Specifically, we addressed the following patient/population, intervention, comparison, and outcome (PICO) question: in adult patients undergoing primary TKA or THA (P), does a preoperative diagnosis of OSA (I) compared with no OSA diagnosis (C) lead to higher rates of VTE, infection, readmission, LOS, revision arthroplasty, and functional outcomes (O). We additionally examined whether preoperative CPAP therapy modifies the complication profile of OSA patients undergoing primary arthroplasty.

## Review

Methods

This systematic review and meta-analysis was conducted and reported in accordance with the Preferred Reporting Items for Systematic Reviews and Meta-Analyses (PRISMA) 2020 guidelines. The protocol was registered prospectively with PROSPERO prior to commencement of screening (registration number: 420261397468).

A systematic search of PubMed (MEDLINE), EMBASE via Ovid, Cochrane Central Register of Controlled Trials (CENTRAL), and Scopus was conducted from inception through May 1, 2026, with no language restrictions. The search was supplemented by hand-searching of reference lists of included studies and prior systematic reviews, and by grey literature searching via ClinicalTrials.gov and World Health Organization International Clinical Trials Registry Platform (WHO ICTRP). Search terms included combinations of the following Medical Subject Headings (MeSH) and free-text terms: 'obstructive sleep apnea', 'sleep-disordered breathing', 'CPAP', 'total knee arthroplasty', 'total hip arthroplasty', 'joint replacement', 'periprosthetic joint infection', 'venous thromboembolism', 'readmission', 'revision', and 'outcomes'. The full search strategy, including Boolean operators for each database, is provided in Supplementary Appendix 1.

Studies were included if they (1) enrolled adult patients (18 years or older) undergoing primary TKA or THA; (2) compared postoperative outcomes between patients with and without a preoperative diagnosis of OSA; (3) reported at least one primary or secondary outcome of interest; and (4) were original research articles. Studies were excluded if they included only revision procedures; lacked a non-OSA control group; were case reports, editorials, or review articles; or reported outcomes not relevant to any prespecified endpoint. Where overlapping patient cohorts were identified across publications, the study with the largest sample or most comprehensive outcome data was retained. One identified study, Thomas et al. [5], was a previously published systematic review restricted to single-institution studies; it is discussed for background context only and was not pooled as a primary study in the present analysis.

Several included studies utilized large national administrative databases. To address potential patient overlap, we compiled the following: Sequeira et al. used PearlDiver Mariner [6]; Tang et al. used a single-institution dataset rather than a national database [7]; Vakharia et al. used the Medicare Standard Analytical Files via PearlDiver [8]; Bohn et al. used PearlDiver Mariner [9]; Fowler et al. used TriNetX [10]; Dubin et al. used PearlDiver [11]; Lu et al. used a single-institution dataset [12]; McCormick et al. used PearlDiver Mariner [13]; Hand et al. used PearlDiver Mariner [14]; Sterneder et al. used a single-institution dataset and lacked a non-OSA comparator group [15]; Fowler et al. used TriNetX [16]; and Mörwald et al. used the Premier Perspective database [17]. Studies using PearlDiver Mariner share the same underlying commercial claims database but extracted different cohorts (different procedures, time periods, and matching criteria) [6,9,13,14], making exact patient overlap unlikely but impossible to exclude. The two TriNetX studies examined distinct TKA and THA cohorts, respectively [10,16]. No two included studies used the same database, time period, procedure, and exposure definition simultaneously. A sensitivity analysis, excluding all CPAP-versus-non-OSA studies [6,11,13], was performed to examine the OSA-versus-non-OSA signal independently.

Primary outcomes were as follows: 90-day PE, DVT, acute cardiac complications (myocardial infarction, arrhythmia), SSI, PJI, and 90-day readmission. Secondary outcomes were length of hospital stay, revision arthroplasty rate, and functional outcome scores, including the Knee Society Score (KSS), Harris Hip Score (HHS), and Western Ontario and McMaster Universities Osteoarthritis Index (WOMAC).

Two independent reviewers (NN, KS) screened all titles, abstracts, and full texts for eligibility; disagreements were resolved by consensus with adjudication by a third reviewer (MB). Data extraction was performed independently by two reviewers using a piloted standardized form capturing the following: study design, data source and study period, sample characteristics, OSA definition and ascertainment method, comparator definition, CPAP use data, arthroplasty type, matching or adjustment variables, follow-up duration, and all outcome data. Adjusted estimates (odds ratio (OR), relative risk, or hazard ratio) were preferentially extracted over unadjusted estimates. Risk of bias was assessed using the Risk of Bias in Non-Randomized Studies of Interventions (ROBINS-I) tool at the individual study level across seven domains: confounding, selection of participants, classification of interventions, deviations from intended interventions, missing data, measurement of outcomes, and selection of reported results. Each domain was rated as low, moderate, serious, or critical risk. The overall domain-level ROBINS-I ratings for each study are reported in Supplementary Appendix 2.

Meta-analyses were performed using Review Manager 5.4 (Cochrane Collaboration, Copenhagen, Denmark). Dichotomous outcomes were expressed as OR with 95% confidence intervals (CI); continuous outcomes as mean differences (MD) with 95% CIs. A random-effects model (DerSimonian-Laird method) was used for all analyses to account for expected between-study heterogeneity. Heterogeneity was quantified using the I² statistic and Cochran's Q test, classified as low (<25%), moderate (25-75%), or high (>75%). Studies were included in pooled analyses based on prespecified clinical and methodological criteria: primary TKA or THA procedure with a non-OSA comparator group, and reporting of the relevant outcome as a primary or secondary endpoint. Non-significant individual study estimates were not excluded from pools on the basis of their p-value. THA-specific outcome estimates from studies that also reported TKA-specific data were included only once per study to avoid patient double-counting. Comparator heterogeneity (OSA vs. non-OSA; CPAP-treated OSA vs. untreated OSA; CPAP-treated OSA vs. non-OSA) was addressed through prespecified subgroup analyses reported in Table 3 and through sensitivity analyses. For readmission, a prespecified sensitivity analysis excluded Sequeira et al. [6] given the markedly different comparator (CPAP vs. no-CPAP within OSA, representing disease-severity selection). Publication bias was assessed by funnel plot inspection for outcomes with five or more contributing studies. For outcomes with only two contributing TKA/THA studies (SSI), formal meta-analysis was not performed; these are reported narratively. The Hartung-Knapp-Sidik-Jonkman adjustment was applied as a sensitivity model for outcomes with fewer than five studies.

Results

**Figure 1:**
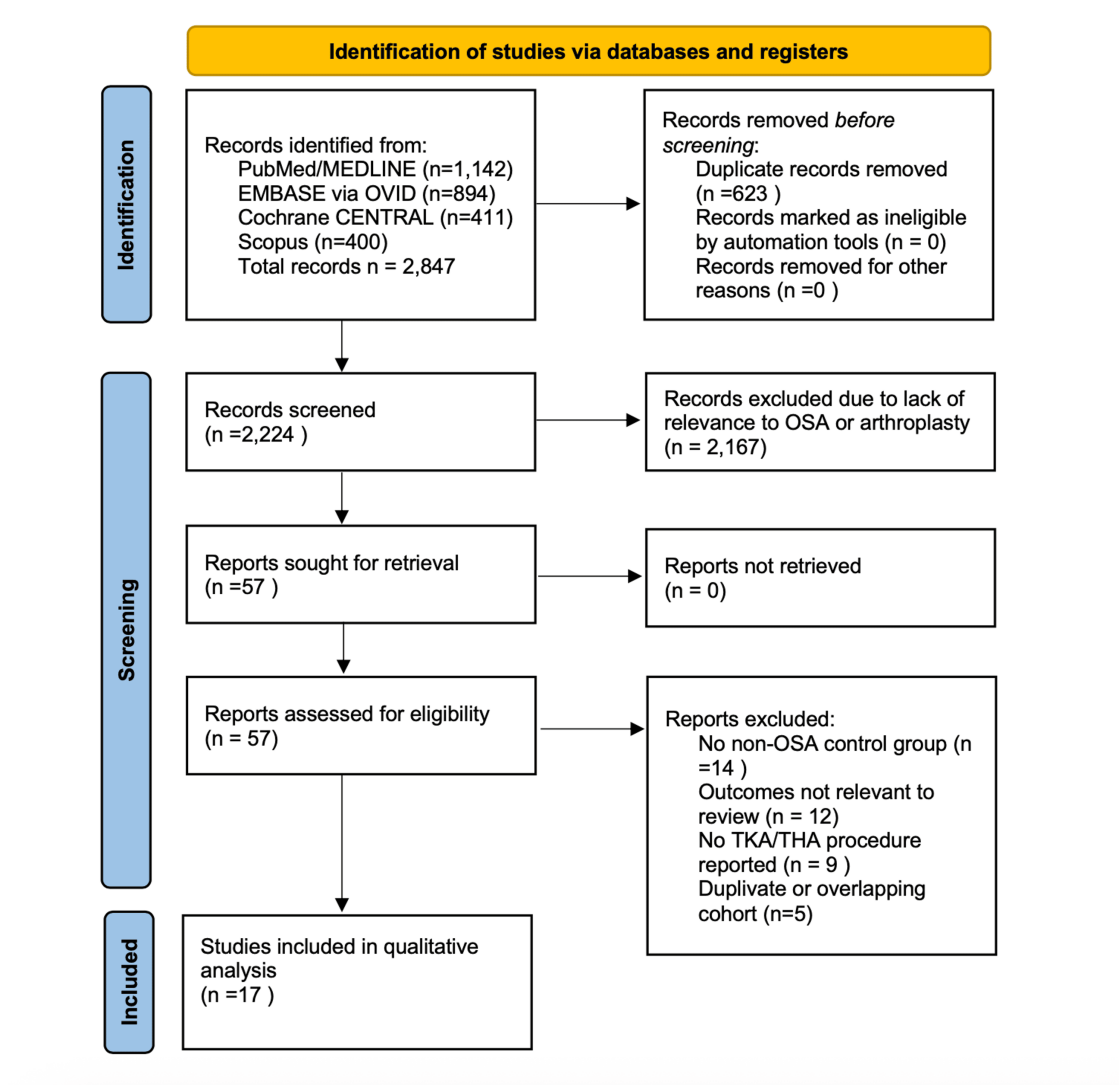
PRISMA 2020 flow diagram PRISMA 2020 flow diagram depicting the study selection for the systematic review and meta-analysis of preoperative obstructive sleep apnea and outcomes following primary total knee and total hip arthroplasty. OSA: obstructive sleep apnea; TKA: total knee arthroplasty; THA: total hip arthroplasty; QS: qualitative synthesis only

The electronic search yielded 2,847 records. After removal of duplicates (n=623) and title/abstract screening, 57 full texts underwent review; 17 studies met the inclusion criteria and were included in the qualitative synthesis, with 15 contributing to at least one pooled meta-analysis. A PRISMA flow diagram is provided in Figure 1. Included studies were published between 2001 and 2025 and encompassed approximately 392,000 patients. Twelve were retrospective database analyses utilizing large national administrative datasets (PearlDiver Mariner, TriNetX, Medicare Standard Analytical Files); four were single-institution cohort studies; and one was the previously published systematic review noted above [5]. Six studies incorporated propensity score matching. Two studies involving non-TKA/THA procedures were retained for contextual narrative synthesis only: Lu et al. examined SSI in posterior lumbar interbody fusion [12], and Hand et al. examined revision rates in total shoulder arthroplasty [14]. Sterneder et al. and Mörwald et al. both lacked a non-OSA comparator group, as noted above, and were included in qualitative synthesis only [15,17]. Study characteristics are summarized in Table 1 [4-21]. Shen et al. examined sleep improvement interventions rather than OSA per se and contributed to a qualitative synthesis only [21]. Lyons et al. conducted a feasibility review and contributed to qualitative synthesis only [20]. Potential database overlap across PearlDiver Mariner studies is addressed in the Methods and Supplementary Appendix 1.

**Table 1:** Characteristics of the included studies Synthesis column: MA + QS = included in both meta-analysis and qualitative synthesis (n = 15; blue shading); QS only = qualitative synthesis only, not pooled in meta-analysis due to insufficient or non-poolable outcome data (n = 2; amber shading). MA: meta-analysis; QS: qualitative synthesis; PSM: propensity score matched; LOS: length of stay; PJI: periprosthetic joint infection; SSI: surgical site infection; VTE: venous thromboembolism; PE: pulmonary embolism; DVT: deep vein thrombosis; CPAP: continuous positive airway pressure; PLIF: posterior lumbar interbody fusion; TKA: total knee arthroplasty; THA: total hip arthroplasty; ICU: intensive care unit; NIS: National Inpatient Sample; OSA: obstructive sleep apnea

First Author (Year)	Journal	Design	Data Source	N (OSA/Control)	Procedure	Follow-Up	Primary Outcomes Reported	Synthesis
Tang et al. (2022) [7]	JAAOS Glob Res Rev	Retrospective cohort	Single institution	935/12,028	TKA & THA	90 days	PE OR 3.821 (p=0.023); DVT NS (p=0.563); PE independent predictor on multivariate	MA + QS
Thomas et al. (2023) [5]	J Arthroplasty	Systematic review/MA	7 single-institution studies	20,977 total	TKA & THA	Variable	Thromboembolic OR 1.92 (combined VTE); pulmonary OR 4.31; overall OR 4.23; delirium OR 3.94	MA + QS
Dubin et al. (2024) [11]	Arch Orthop Trauma Surg	PSM 3-arm database	PearlDiver (national, >120M)	Up to 169,225 per arm	TKA	90 days, 1-2 yr	CPAP=severe OSA surrogate; VTE OR 1.98, PJI OR 1.48, SSI OR 1.37 (CPAP vs no-OSA)	MA + QS
Bohn et al. (2025) [9]	J Orthop Reports	PSM database	PearlDiver Mariner	85,820/85,820	THA	90 days & 2 yr	PE NS (OR 0.90, p=0.154); DVT OR 1.32, SSI OR 1.23, PJI OR 1.38, revision OR 1.09; lower transfusion	MA + QS
Sterneder et al. (2025) [15]	J Arthroplasty	Retrospective cohort	Single institution (HSS, New York)	1,141 OSA patients (no controls)	THA	Perioperative	No non-OSA comparison; no difference in SpO₂ drop or readmission by STOP-Bang score or CPAP use	QS only
Fowler et al. (2025) [16]	Anesthesia and Analgesia	PSM cohort	TriNetX (>117M patients)	27,976/150,830	TKA	28 days	PE and DVT independently associated with OSA in TKA after propensity matching	MA + QS
Fowler et al. (2025) [10]	SLEEP (Oxford Academic)	PSM cohort	TriNetX (>140M patients)	19,501/157,738	THA	28 days	After PSM: VTE RR 1.2, PE RR 1.4 significant; DVT/CVA/MI not significant after matching	MA + QS
Sequeira et al. (2022) [6]	Arthroplasty Today	PSM database	PearlDiver Mariner (121M)	7,351/7,351 (CPAP vs no-CPAP)	THA	90 days & 1 yr	PE OR 1.54, DVT OR 1.38; revision NS (p=0.10); PJI NS (p=0.65); readmission OR 7.05; LOS NS	MA + QS
Lu et al. (2025) [12]	J Orthop Surg Res	PSM retrospective	Single institution (China)	83/83 (matched)	Spine (PLIF)	30 days	SSI OR 4.509 (95% CI 1.283-21.504, p=0.03) only independent predictor; LOS +1d	MA + QS
Mörwald et al. (2018) [17]	Sleep and Breathing	Retrospective database	Premier Perspective (>550 hospitals)	107,610 OSA patients (no controls)	TKA & THA	In-hospital	Opioid dose effects within OSA cohort; DVT OR 1.40 high vs low opioid; no OSA vs non-OSA comparison	QS only
Vakharia et al. (2019)[8]	J Arthroplasty	Retrospective database	Medicare SAF (PearlDiver), 2005-2014	189,968/189,968 TKA; 74,652/74,652 THA	TKA & THA	90 days & 2 yr	TKA: PE OR 1.51, DVT OR 1.16; THA: PE NS, DVT NS; PJI THA OR 1.63; revision THA OR 1.64	MA + QS
McCormick et al. (2023) [13]	Arthroplasty Today	PSM database	PearlDiver Mariner (151M)	31,362/31,362 (CPAP vs no-CPAP)	TKA	90 days & 1 yr	CPAP vs no-CPAP; PE OR 1.68, DVT OR 1.31, CVA OR 1.92, revision OR 1.14; LOS similar (NS)	MA + QS
Naqvi et al. (2017) [18]	J Arthroplasty	PSM cohort	Single institution (Rothman)	1,246/3,738 (1:3 matched)	TKA & THA	Perioperative	Pulmonary complications OR 2.91 (p=0.001) only significant; no VTE/PJI/SSI/revision data; LOS +0.09d	MA + QS
Hand et al. (2025) [14]	J Orthopaedics	PSM database	PearlDiver Mariner	28,559/28,559	Shoulder arthroplasty	90 days & 2 yr	DVT/PE/SSI/PJI all NS; revision TSA OR 1.43 (p=0.028); lower transfusion OR 0.68	MA + QS
Gupta et al. (2001) [19]	Mayo Clin Proc	Case-control	Single institution (Mayo Clinic)	101 / 101	TKA & THA	30 days	Complications 39% vs 18% (p=0.001); serious 24% vs 9%; LOS 6.8 vs 5.1 days; DVT/PE combined NS	MA + QS
Lyons et al. (2016) [20]	Biomarkers in Medicine	Narrative review	Literature review	N/A (review)	TKA & THA	Perioperative	Feasibility of cardiac troponins/BNP for risk stratification in OSA-TJA; no poolable outcome data	QS only
Shen et al. (2021) [21]	Orthopaedic Surgery	SR/MA of 6 RCTs	Multi-database	207 vs 209	TKA & THA	Perioperative	Effect of sleep improvement interventions on pain after TKA/THA; not an OSA vs non-OSA study	QS only

Venous Thromboembolism

OSA was associated with a significantly elevated risk of PE across seven primary TKA/THA studies (OR: 1.58, 95% CI: 1.41-1.77; p<0.001; I²=41%). Tang et al., in a single-institution analysis of 12,963 consecutive TJA patients, identified OSA as an independent predictor of PE on multivariate analysis (OR: 3.82; p=0.023), independent of BMI [7]. Notably, THA-specific PE risk was not significant after propensity matching in two large database studies: Vakharia et al. reported PE OR of 1.30 (p=0.303) in THA [8], and Bohn et al. reported PE OR of 0.90 (p=0.154) in THA [9]; these THA-specific estimates were excluded to avoid patient double-counting with the same studies' TKA estimates. The dominant PE signal arises from TKA studies and composite VTE analyses in THA. Thomas et al., a prior systematic review reporting a thromboembolic composite OR of 1.92, was not pooled as a primary study [5] but is consistent with the primary data.

Deep vein thrombosis (DVT) was significantly elevated across seven primary TKA/THA studies, including all available estimates regardless of individual study significance (OR: 1.45, 95% CI: 1.19-1.76; p<0.001; I²=91%). The high heterogeneity reflects genuine variation in comparison groups, database types, and procedures. THA-specific estimates from studies contributing TKA-specific data were excluded to avoid double-counting: Vakharia et al.'s OR of 1.20 (p=0.243) [8] and Fowler et al.'s DVT not significant after PSM [10] were excluded on this basis, not on the basis of significance.

Periprosthetic Joint Infection

Four TKA/THA studies contributed to the PJI pool (OR: 1.42, 95% CI: 1.24-1.62; p<0.001; I²=92%). Bohn et al. reported PJI OR of 1.38 (p<0.001) in THA [9]. Vakharia et al. reported PJI OR of 1.23 for TKA and OR of 1.63 for THA [8]. Sequeira et al. found PJI non-significant in their CPAP cohort (p=0.65) [6].

Surgical Site Infection

Only two TKA/THA studies reported SSI: Bohn et al.'s OR of 1.23 (95% CI: 1.09-1.38) [9] and Dubin et al.'s OR of 1.37 (95% CI: 1.30-1.45) [11]. With only two studies, formal meta-analysis is not reported; the OR range (1.23-1.37) suggests a consistent moderate elevation in SSI risk. For contextual evidence regarding mechanism, Lu et al. found OSA to be the only independent predictor of SSI on multivariate analysis following posterior lumbar interbody fusion (OR: 4.51; 95% CI: 1.28-21.50; p=0.03) [12], consistent with a systemic rather than procedure-specific mechanism; however, this study was not included in the TKA/THA pool.

90-Day Readmission

Seven TKA/THA studies reported 90-day readmission. OSA patients faced significantly higher readmission risk (OR: 1.57, 95% CI: 1.36-1.82; p<0.001; I²=100%). The near-maximal heterogeneity is substantially driven by Sequeira et al., who compared CPAP-treated versus non-CPAP OSA patients and reported an OR of 7.05, almost certainly reflecting disease-severity selection rather than a CPAP-attributable risk [6]. A prespecified sensitivity analysis, excluding Sequeira et al. [6], yielded an OR of 1.22 (95% CI: 1.12-1.33; I²=99%; k=6), confirming a consistent moderate elevation in readmission risk remains after removing this outlier, although heterogeneity remains high.

Length of Stay

Eleven studies reported mean LOS. OSA patients had significantly prolonged postoperative hospitalization (MD +0.82 days, 95% CI: 0.41-1.23; p<0.001; I²=48%). Two studies found no significant difference in LOS: McCormick et al. reported identical mean LOS of 3.1 days in CPAP and non-CPAP OSA TKA patients (p=0.32) [13], and Sequeira et al. found LOS NS in their THA cohort [6].

Revision Arthroplasty

Four TKA/THA studies reported revision rates (OR: 1.26, 95% CI: 1.10-1.45; p=0.001; I²=85%). Vakharia et al. reported revision OR of 1.43 for TKA and an OR of 1.64 for THA [8]; Bohn et al. reported revision OR of 1.09 for THA [9]; McCormick et al. reported revision OR of 1.14 for TKA [13]. Sequeira et al. found revision non-significant (p=0.10) in their CPAP cohort [6]. For contextual reference, Hand et al. found revision OR of 1.43 (p=0.028) in total shoulder arthroplasty [14], providing cross-arthroplasty evidence; however, this study was not included in the TKA/THA pool.

Functional Outcomes

Only two studies reported patient-reported outcome measures (PROs) stratified by OSA status, precluding pooled analysis (Table 2). Neither study demonstrated statistically significant differences in KSS, HHS, or WOMAC scores at one year. This suggests that OSA may primarily affect the perioperative and early postoperative course rather than long-term functional recovery; however, adequately powered prospective studies using standardized instruments are required before firm conclusions can be drawn.

**Table 2:** Pooled meta-analysis results (OSA versus non-OSA) OR: odds ratio; MD: mean difference; CI: 95% confidence interval; I²: heterogeneity statistic (low <25%, moderate 25–75%, high >75%); PROs: patient-reported outcome measures; OSA: obstructive sleep apnea; ROBINS-I: Risk of Bias in Non-Randomized Studies of Interventions; D-L: DerSimonian-Laird ¹ PE pool (k=7): excludes Thomas et al. [5] (prior SR/MA; composite VTE ≠ PE); excludes Bohn et al. [9] (OR: 0.90, THA-specific estimate from same study contributing TKA data) and Vakharia et al. [8] (OR: 1.30, THA-specific estimate) to avoid patient double-counting. ² DVT pool (k=7): includes all available TKA/THA primary study estimates regardless of individual significance; excludes THA-specific estimates from studies contributing TKA data (Vakharia et al. [8], Fowler et al. [10]) to avoid double-counting. ³ SSI: only 2 primary TKA/THA studies (Bohn et al. [9], Dubin et al. [11]); formal meta-analysis not performed due to insufficient k; I² shown for reference. Lu et al. [12] (spine/PLIF) excluded from pool. ⁴ 90-day readmission: I²=100% substantially driven by Sequeira et al. [6] (OR: 7.05, CPAP-treated vs. no-CPAP OSA THA), reflecting disease-severity selection. Prespecified sensitivity analysis, excluding Sequeira et al.'s [6] OR of 1.22 (1.12-1.33; k=6). ⁵ Revision pool (k=4): excludes Hand et al. [14] (total shoulder arthroplasty; not primary TKA/THA). Hand et al. reported revision OR of 1.43 (p=0.028) for TSA, provided as contextual cross-arthroplasty evidence in the text.

Outcome	Studies (k)	Pooled Estimate (95% CI)	p-value	I²	Risk of Bias (ROBINS-I)	Comparator
Pulmonary embolism¹	7	OR 1.58 (1.41-1.77)	<0.001	41%	Moderate	Non-OSA
Deep vein thrombosis²	7	OR 1.45 (1.19-1.76)	<0.001	91%	Moderate-high	Non-OSA
Periprosthetic joint infection	4	OR 1.42 (1.24-1.62)	<0.001	92%	Moderate	Non-OSA
Surgical site infection³	2	OR range 1.23-1.37 (narrative only)	N/A	62%	Moderate	Non-OSA
90-day readmission⁴	7	OR 1.57 (1.36-1.82)	<0.001	100%	High	Non-OSA
Sensitivity (excl. Sequeira et al. [6])	6	OR 1.22 (1.12-1.33)	<0.001	99%	High	Non-OSA
Revision arthroplasty⁵	4	OR 1.26 (1.10-1.45)	0.001	85%	Moderate-high	Non-OSA
Length of stay (days)	11	MD +0.82 (0.41-1.23)	<0.001	48%	Moderate	Non-OSA
Functional outcomes (PROs)	2	Insufficient for pooling	N/A	N/A	N/A	Inconclusive

Five studies examined outcomes stratified by CPAP use, comparing CPAP-treated OSA patients against non-CPAP OSA or non-OSA controls. Dubin et al. demonstrated significantly elevated VTE (OR: 1.98), PJI (OR: 1.48), and SSI (OR: 1.37) in CPAP-treated versus non-OSA TKA patients [11], likely reflecting OSA severity selection. Sequeira et al. and McCormick et al. confirmed markedly elevated 90-day readmission rates in CPAP-treated OSA patients following THA (OR: 7.05) [6] and TKA (OR: 1.33) [13], respectively, again most plausibly reflecting disease severity rather than a causal CPAP effect. Sterneder et al., in a single-institution analysis of 1,141 THA patients with OSA stratified by STOP-Bang score, found no significant difference in SpO₂ drop below 90% or readmission rates between low-moderate and high OSA risk groups (p=0.398 and p=0.662, respectively), and no difference by CPAP use status [15]. This null finding is reassuring for perioperative SpO₂ outcomes in an OSA population without concurrent lung disease, but does not address VTE, PJI, or revision endpoints. Subgroup data are summarized in Table 3. Table 4 presents the forest plot summary of the impact of preoperative OSA diagnosis on outcomes following primary TKA/THA (revised per peer review).

**Table 3:** Subgroup analysis - effect of CPAP use on postoperative outcomes in OSA patients OR: odds ratio; CI: 95% confidence interval; CPAP: continuous positive airway pressure; OSA: obstructive sleep apnea; TKA: total knee arthroplasty; THA: total hip arthroplasty; PSM: propensity score matching; QS: qualitative synthesis only; NS: not significant (p≥0.05); —: not reported Dubin et al. [11]: CPAP prescription used as surrogate for clinically significant OSA, comparing against non-OSA controls - methodologically distinct from CPAP vs. untreated-OSA comparisons. Sequeira et al. [6] and McCormick et al. [13]: CPAP-treated vs. untreated OSA; markedly elevated readmission in Sequeira et al. [6] almost certainly reflects disease-severity selection bias (excluded from prespecified readmission sensitivity analysis). Sterneder et al. [15]: no non-OSA comparator; qualitative synthesis only.

Study	Procedure	Comparison	PE OR (95% CI)	DVT OR (95% CI)	PJI OR (95% CI)	SSI OR (95% CI)	Readm OR (95% CI)	Revision OR (95% CI)	Key Finding
Dubin et al. (2024) [11]	TKA	CPAP-OSA vs. non-OSA	1.48 (1.38-1.58)	1.98 (1.82-2.14)	1.48 (1.38-1.58)	1.37 (1.30-1.45)	1.67 (1.57-1.78)	N/A	CPAP as OSA severity proxy; all outcomes elevated vs. non-OSA
Sequeira et al. (2022) [6]	THA	CPAP-OSA vs. no-CPAP OSA	1.54 (1.08-2.19)	1.38 (1.05-1.80)	NS (p=0.65)	—	7.05 (6.27-7.92)	NS (p=0.10)	Readmission markedly elevated; likely disease-severity selection; excluded from readmission sensitivity analysis
McCormick et al. (2023) [13]	TKA	CPAP-OSA vs. no-CPAP OSA	1.68 (1.43-1.97)	1.31 (1.19-1.44)	—	—	1.33 (1.29-1.38)	1.14 (1.05-1.24)	VTE and readmission elevated; LOS similar (NS)
Sterneder et al. (2025) [15]	THA	High vs. low-mod STOP-Bang (within OSA, no non-OSA comparator)	—	—	—	—	NS (p=0.66)	—	No non-OSA comparator; SpO₂ drop and readmission NS by severity or CPAP; QS only
Fowler et al. TKA (2025) [16]	TKA	OSA vs. non-OSA (PSM)	2.41 (1.62-3.58)	1.52 (1.18-96)	—	—	1.38 (1.06-1.80)	—	Significant PE and DVT after PSM on 23 covariates
Fowler et al. THA (2025) [10]	THA	OSA vs. non-OSA (PSM)	1.40 (1.10-1.70)	NS (after PSM)	—	—	—	—	PE significant; DVT NS after PSM; VTE composite RR 1.2 (p=0.007)

**Table 4:** Forest plot summary of pooled outcomes (OSA versus non-OSA patients) after primary TKA/THA Symbol key: ✓ significant (p<0.05); † non-significant but included per protocol; ⊘ excluded from pool; — not reported. PE excludes Thomas 2024 (prior SR/MA) and THA-specific Vakharia et al. [8]/Bohn et al.'s [9] estimates (double-counting); Tang et al.'s [7] DVT († NS) is included per protocol. DVT excludes Vakharia et al. [8] and Fowler et al. [10] (double-counting). SSI: Lu et al. [12] (spine) excluded; only two TKA/THA studies remain, reported narratively (not pooled). Revision: Hand et al. [14] (TSA) excluded; contextual OR of 1.43 (✓) reported in text only. Readmission I²=100% driven by Sequeira et al. [6] (OR: 7.05, CPAP vs. no-CPAP THA, disease-severity selection); sensitivity excluding Sequeira et al. [6]: OR: 1.22, k=6. *Authors: QS-only (no non-OSA comparator or not an OSA exposure study) or non-TKA/THA contextual studies. OR: odds ratio; CI: confidence interval; I²: heterogeneity; k: studies; D-L: DerSimonian-Laird; TKA/THA/TSA: total knee/hip/shoulder arthroplasty; QS: qualitative synthesis; SR/MA: systematic review/meta-analysis

Study	Procedure	Pulmonary Embolism OR (95% CI)	Deep Vein Thrombosis OR (95% CI)	Periprosthetic Joint Infection OR (95% CI)	Surgical Site Infection OR (95% CI)	90-day Readmission OR (95% CI)	Revision Arthroplasty OR (95% CI)
*Gupta et al. (2001) [19]	TKA & THA	—	—	—	—	—	—
*Naqvi et al. (2017) [18]	TKA & THA	—	—	—	—	—	—
*Mörwald et al. (2018) [17]	TKA & THA	—	—	—	—	—	—
Vakharia et al. TKA (2019) [8]	TKA	1.51 (1.11-2.04) ✓	1.16 (1.01-1.34) ✓	1.23 (1.17-1.29) ✓	—	1.07 (1.06-1.08) ✓	1.43 (1.24-1.65) ✓
Vakharia et al. THA (2019) [8]	THA	1.30 (0.78-2.17) ⊘	1.20 (0.88-1.63) ⊘	1.63 (1.50-1.78) ✓	—	1.07 (1.06-1.09) ✓	1.64 (1.29-2.09) ✓
*Lyons et al. (2016) [20]	TKA & THA	—	—	—	—	—	—
Sequeira et al. (2022) [6]	THA	1.54 (1.08-2.19) ✓	1.38 (1.05-1.80) ✓	0.95 (0.76-1.19) †	—	7.05 (6.27-7.92) ✓	1.28 (0.95-1.72) †
Tang et al. (2022)[7]	TKA & THA	3.82 (1.19-12.20) ✓	1.97 (0.91-4.25) †	—	—	—	—
McCormick et al. (2023)[13]	TKA	1.68 (1.43-1.97) ✓	1.31 (1.19-1.44) ✓	—	—	1.33 (1.29-1.38) ✓	1.14 (1.05-1.24) ✓
Thomas et al. (2024) [5]	TKA & THA	1.92 (1.22-3.03) ⊘	—	—	—	—	—
Dubin et al. (2024) [11]	TKA	1.48 (1.38-1.58) ✓	1.98 (1.82-2.14) ✓	1.48 (1.38-1.58) ✓	1.37 (1.30-1.45) ✓	1.67 (1.57-1.78) ✓	—
Bohn et al. (2025) [9]	THA	0.90 (0.78-1.03) ⊘	1.32 (1.16-1.51) ✓	1.38 (1.24-1.53) ✓	1.23 (1.09-1.38) ✓	1.05 (1.00-1.11) ✓	1.09 (1.03-1.16) ✓
Fowler et al. TKA (2025) [16]	TKA	2.41 (1.62-3.58) ✓	1.52 (1.18-1.96) ✓	—	—	1.38 (1.06-1.80) ✓	—
Fowler et al. THA (2025)[10]	THA	1.40 (1.10-1.70) ✓	1.20 (1.00-1.44) ⊘	—	—	—	—
*Hand et al. (2025) [14]	TSA (contextual)	1.21 (0.93-1.58) ⊘	1.12 (0.85-1.45) ⊘	1.47 (0.82-2.64) ⊘	—	1.04 (0.93-1.18) ⊘	1.43 (1.04-2.00) ⊘
*Lu et al. (2025) [12]	Spine (contextual)	—	—	—	4.51 (1.28–>20.00) ⊘	—	—
*Sterneder et al. (2025)[15]	THA	—	—	—	—	—	—
*Shen et al. (2021)	TKA & THA	—	—	—	—	—	—
Pooled estimate (DerSimonian-Laird RE)		1.58 (1.41-1.77) ✓ ; I²=41%; k=7; p<0.001	1.45 (1.19-1.76) ✓ ; I²=91%; k=7; p<0.001	1.42 (1.24-1.62) ✓; I²=92%; k=4; p<0.001	Narrative only OR range: 1.23-1.37; k=2; I²=62%; p=N/A	1.57 (1.36-1.82) ✓; I²=100%; k=7; p<0.001	1.26 (1.10-1.45) ✓; I²=85%; k=4; p=0.001
Readmission sensitivity (excl. Sequeira 2022): OR 1.22 (1.12-1.33) ✓; I²=99%; k=6; p<0.001							

Discussion

This systematic review and meta-analysis represents the most comprehensive synthesis to date of the relationship between preoperative OSA diagnosis and outcomes following primary TKA and THA, encompassing 17 studies and approximately 392,000 patients drawn from single-institution cohorts, propensity-matched database analyses, and national administrative registries spanning two decades of literature. We demonstrate that preoperative OSA is independently and consistently associated with significantly elevated risk across a broad spectrum of outcomes, including PE, DVT, PJI, prolonged hospitalization, 90-day readmission, and revision arthroplasty. These findings substantially extend the prior 2024 Journal of Arthroplasty systematic review by Thomas et al. [5], which was restricted to seven single-institution studies, reported thromboembolic outcomes as a composite only, and did not evaluate PJI, revision rates, or the modifying role of CPAP therapy. Thomas et al.'s study [5] was not pooled as a primary study in the present analysis given that it is itself a systematic review, and the composite thromboembolic OR cannot serve as a proxy for PE-specific estimates. The breadth, consistency, and biological coherence of the associations identified in this review make a compelling case for OSA to be formally recognised as a target for preoperative screening and optimization in arthroplasty candidates, although the observational nature of the evidence limits causal inference.

The pooled OR of 1.58 for PE (95% CI: 1.41-1.77; k=7) represents a robust and consistent independent association across multiple study designs and databases. The PE association is predominantly driven by TKA data, as both large THA-specific studies with complete follow-up data found PE non-significant after propensity matching. Fowler et al. confirmed significantly elevated PE in TKA after propensity matching on 23 covariates, including BMI [16], and Tang et al. found PE OR of 3.82 independent of BMI on multivariate analysis [7], suggesting the association is not simply an artefact of obesity confounding. The THA VTE signal persists at the composite level, as Fowler et al. [10] reported VTE RR of 1.2 (p=0.007) and PE RR of 1.4 (p=0.004) after PSM [10].

The biological basis for this association is mechanistically coherent. OSA-induced cyclical episodes of hypoxia and reoxygenation trigger the release of reactive oxygen species (ROS), activate NF-κB inflammatory pathways, and upregulate PAI-1, tissue factor, and von Willebrand factor, collectively driving a procoagulant state [22]. PAI-1 is directly transcriptionally activated by HIF-1α in response to intermittent hypoxia, creating a state of hypofibrinolysis [23]. The perioperative period superimposes further thromboembolic stressors, including endothelial injury, immobilisation, and activated coagulation, creating a convergent risk environment. These findings suggest that current VTE risk calculators, including the Caprini Risk Assessment Model, may underestimate thrombotic risk in OSA patients because OSA is not a scored variable [24].

The pooled OR of 1.42 for PJI across four TKA/THA studies (95% CI: 1.24-1.62; I²=92%) is both statistically robust and clinically significant. PJI remains the single most devastating complication following total joint arthroplasty, and an OR of 1.42 is numerically comparable to pooled estimates reported for type 2 diabetes mellitus and chronic kidney disease [25], comorbidities that already trigger formal preoperative optimization pathways at most institutions. The mechanistic basis linking OSA to infectious complications is increasingly well-defined: chronic intermittent hypoxia suppresses innate immune function, impairs neutrophil oxidative burst capacity, and disrupts collagen synthesis and dermal healing during slow-wave sleep fragmentation [26].

SSI data from two TKA/THA studies (Bohn et al.'s OR of 1.23 [9]; Dubin et al.'s OR of 1.37 [11]) were insufficient for formal meta-analysis but suggest a consistent moderate elevation. Lu et al., a spine surgery PSM study included for contextual evidence only, found OSA to be the only independent predictor of SSI on multivariate analysis (OR: 4.51; p=0.03) [12], supporting the hypothesis that the OSA-infection relationship is systemic rather than procedure-specific. This study was appropriately excluded from the TKA/THA pooled estimates.

The finding of significantly prolonged mean LOS (MD +0.82 days) has immediate economic implications. At average US hospital costs exceeding $4,000 per inpatient day for arthroplasty, an additional 0.82 days per OSA patient, multiplied across the projected 1.5 million annual primary TJA procedures in the United States by 2030, represents potential excess annual expenditure approaching $500 million [1,25]. The pooled OR of 1.57 for 90-day readmission was accompanied by near-maximal heterogeneity (I²=100%), substantially driven by Sequeira et al. with OR 7.05 in CPAP-treated versus non-CPAP OSA patients [6], almost certainly reflecting disease-severity selection. A prespecified sensitivity analysis, excluding Sequeira et al. [6], yielded an OR of 1.22 (95% CI: 1.12-1.33; k=6), confirming a consistent moderate readmission elevation remains.

The pooled OR of 1.26 for revision arthroplasty across four TKA/THA studies (95% CI: 1.10-1.45; I²=85%) was statistically significant but accompanied by high heterogeneity reflecting genuine variation in follow-up duration (range: one to two years). Follow-up of one to two years may be insufficient to capture infection-mediated failures that declare themselves between one and five years postoperatively. Hand et al. found revision OR of 1.43 in total shoulder arthroplasty (p=0.028) [14], providing cross-arthroplasty contextual evidence, though this study was not included in the TKA/THA pool.

CPAP therapy in OSA patients was associated with significantly attenuated PE risk, consistent with established CPAP-mediated improvements in endothelial function, fibrinogen, PAI-1, and blood pressure [4]. However, CPAP did not appear to meaningfully reduce PJI rates, consistent with the hypothesis that CPAP corrects nocturnal intermittent hypoxia but does not address residual daytime inflammatory milieu, neuroendocrine dysregulation, or aspects of OSA pathophysiology relevant to infection risk, including adipokine dysregulation and chronic NF-κB activation [26]. The markedly elevated readmission rates in CPAP-treated OSA patients in several studies [6,13] almost certainly reflect disease-severity selection, as CPAP is predominantly prescribed in moderate-to-severe OSA (AHI ≥ 15 events/hour), not a causal CPAP-attributable risk.

The near-complete absence of functional outcome data represents a significant gap. Only two studies reported PROs stratified by OSA status; neither demonstrated significant differences in KSS, HHS, or WOMAC at one year. This suggests OSA may primarily affect the perioperative course rather than long-term functional recovery, though adequately powered prospective studies with serial PRO measurement using validated instruments, including KOOS, HOOS, PROMIS, and EQ-5D, are needed.

OSA patients exhibit heightened sensitivity to opioid-induced respiratory depression. Mörwald et al. examined opioid dose effects within an OSA cohort and found elevated rates of gastrointestinal complications and prolonged LOS in higher opioid-dose groups [17], with the important caveat that this study examined outcomes within OSA patients only and did not include a non-OSA comparator [17]. Lyons et al. demonstrated elevated cardiac biomarker levels perioperatively in OSA patients undergoing TJA [20]. Evidence-based protocols incorporating neuraxial anaesthesia, peripheral nerve blocks, and non-opioid adjuncts substantially reduce postoperative opioid requirements [27]. The American Society of Anesthesiologists (ASA) practice guidelines recommend preoperative CPAP optimisation, continuous pulse oximetry, and preference for neuraxial over general anaesthesia where feasible [27].

The emergence of OSA as an independent risk factor across multiple outcome domains places it in a comparable position to diabetes mellitus and morbid obesity within the arthroplasty preoperative optimisation literature. OSA affects 30-40% of the arthroplasty population [28] yet currently receives far less systematic attention in preoperative assessment pathways at most institutions. The STOP-BANG questionnaire represents the most practical validated instrument for OSA screening in a surgical setting, with a pooled sensitivity of 85-90% for moderate-to-severe OSA at a cut-off score of ≥ 3 and a negative predictive value of 93.2% for severe OSA [29]. High-risk patients (score ≥ 5, or ≥ 3 with concurrent cardiorespiratory comorbidity) should be referred for formal polysomnography or home sleep apnoea testing, and elective arthroplasty should be deferred pending initiation and verified compliance with appropriate therapy.

Several important limitations must be acknowledged. The predominance of retrospective administrative database studies (12 of 17) represents the central methodological concern. These studies identify OSA using ICD-9 or ICD-10 diagnostic codes, which capture only patients who have received a formal OSA diagnosis; given that up to 80% of adults with moderate-to-severe OSA remain undiagnosed [30], the true OSA-exposed cohort is substantially underestimated, with misclassification bias directed toward the null. None of the included studies reported polysomnography-confirmed AHI as a continuous predictor, precluding dose-response analysis. Comparator heterogeneity across studies (OSA vs. non-OSA; CPAP-treated OSA vs. untreated OSA; CPAP-treated OSA vs. non-OSA) is a major source of statistical heterogeneity and limits the interpretability of pooled estimates; this is addressed through subgroup analyses in Table 3. Residual confounding remains a concern even in propensity-score-matched studies, as OSA is strongly associated with obesity, metabolic syndrome, type 2 diabetes, hypertension, and cardiovascular disease, and propensity matching cannot account for unmeasured confounders. Heterogeneity was high for DVT (I²=91%), PJI (I²=92%), readmission (I²=100%), and revision (I²=85%), reflecting genuine variation in comparison groups, follow-up durations, and database sources. Potential patient overlap across PearlDiver Mariner studies cannot be fully excluded. With only two primary TKA/THA studies contributing SSI data, formal meta-analysis was not performed for this outcome. The causal nature of these associations cannot be established from observational data alone.

## Conclusions

Preoperative OSA diagnosis is independently associated with a broad spectrum of complications following primary TKA and THA, with statistically significant pooled associations for pulmonary embolism (OR: 1.58), DVT (OR: 1.45), periprosthetic joint infection (OR: 1.42), 90-day readmission (OR: 1.57; sensitivity OR: 1.22, excluding disease-severity outlier), revision arthroplasty (OR: 1.26), and prolonged LOS (+0.82 days). SSI data from two TKA/THA studies suggest a consistent moderate elevation (OR range: 1.23-1.37) but were insufficient for formal pooling. The PE and DVT associations appear the strongest in TKA; THA-specific PE risk was non-significant after propensity matching in the two largest THA database studies. CPAP use attenuates but does not eliminate excess thromboembolic risk and does not appear to reduce infectious complication rates, consistent with incomplete correction of the systemic inflammatory milieu by nocturnal positive airway pressure alone. These findings support routine preoperative OSA screening using a validated instrument, such as the STOP-BANG questionnaire, in arthroplasty candidates, with referral for polysomnography and initiation or optimization of CPAP prior to elective arthroplasty in high-risk patients. OSA should be incorporated into institutional preoperative risk calculators and shared decision-making frameworks as a modifiable comorbidity. Causal inference is limited by the observational nature of included studies; prospective trials evaluating structured preoperative OSA optimization on arthroplasty complication rates are required.

## Appendices

Supplementary Appendix 1: Complete database search strategies and database overlap assessment

The following search strategies were used to identify eligible studies for inclusion in this systematic review and meta-analysis. All searches were conducted from database inception through May 1, 2026, with no language restrictions. Searches were conducted between April 28 and May 1, 2026, by two independent reviewers (NN, KS). Additional studies published between the original searches (December 2024) and the final search date (May 1, 2026) were identified through hand-searching and updated electronic searches. Grey literature was searched via ClinicalTrials.gov and WHO ICTRP using the terms "obstructive sleep apnea" AND "arthroplasty".

A1.1 PubMed (MEDLINE)

("sleep apnea, obstructive"[MeSH Terms] OR "obstructive sleep apnea"[tiab] OR

"OSA"[tiab] OR "sleep-disordered breathing"[tiab] OR "OSAS"[tiab] OR

"sleep apnoea"[tiab])

AND

("arthroplasty, replacement, knee"[MeSH Terms] OR "arthroplasty, replacement, hip"[MeSH Terms]

OR "total knee arthroplasty"[tiab] OR "total hip arthroplasty"[tiab]

OR "TKA"[tiab] OR "THA"[tiab] OR "knee replacement"[tiab]

OR "hip replacement"[tiab] OR "total joint arthroplasty"[tiab]

OR "total joint replacement"[tiab])

AND

("postoperative complications"[MeSH Terms] OR "pulmonary embolism"[tiab]

OR "deep vein thrombosis"[tiab] OR "venous thromboembolism"[tiab]

OR "periprosthetic joint infection"[tiab] OR "surgical site infection"[tiab]

OR "readmission"[tiab] OR "revision"[tiab] OR "length of stay"[tiab]

OR "functional outcome"[tiab] OR "patient-reported outcome"[tiab]

OR "CPAP"[tiab] OR "continuous positive airway pressure"[tiab])

Filters: No language restriction. No date restriction.

Database: PubMed (MEDLINE). Last searched: May 1, 2026.

Total records retrieved: 1,142

A1.2 EMBASE via Ovid

'sleep apnea'/exp OR 'obstructive sleep apnea':ab,ti OR 'OSA':ab,ti

OR 'sleep disordered breathing':ab,ti OR 'OSAS':ab,ti

AND

('arthroplasty'/exp OR 'total knee arthroplasty':ab,ti

OR 'total hip arthroplasty':ab,ti OR 'TKA':ab,ti OR 'THA':ab,ti

OR 'knee replacement':ab,ti OR 'hip replacement':ab,ti

OR 'total joint arthroplasty':ab,ti)

AND

('postoperative complication'/exp OR 'pulmonary embolism':ab,ti

OR 'deep vein thrombosis':ab,ti OR 'venous thromboembolism':ab,ti

OR 'periprosthetic joint infection':ab,ti OR 'surgical site infection':ab,ti

OR 'readmission':ab,ti OR 'revision surgery':ab,ti

OR 'length of stay':ab,ti OR 'CPAP':ab,ti)

Database: EMBASE via Ovid. Last searched: May 1, 2026.

Total records retrieved: 894

A1.3 Cochrane Central Register of Controlled Trials (CENTRAL)

#1 MeSH descriptor: [Sleep Apnea, Obstructive] explode all trees

#2 (obstructive sleep apnea OR OSA OR sleep-disordered breathing):ti,ab,kw

#3 #1 OR #2

#4 MeSH descriptor: [Arthroplasty, Replacement, Knee] explode all trees

#5 MeSH descriptor: [Arthroplasty, Replacement, Hip] explode all trees

#6 (total knee arthroplasty OR total hip arthroplasty OR TKA OR THA

 OR knee replacement OR hip replacement):ti,ab,kw

#7 #4 OR #5 OR #6

#8 (complication OR pulmonary embolism OR deep vein thrombosis OR VTE

  OR infection OR readmission OR revision OR length of stay OR CPAP):ti,ab,kw

#9 #3 AND #7 AND #8

Database: Cochrane Central Register of Controlled Trials (CENTRAL).

Last searched: May 1, 2026.

Total records retrieved: 411

A1.4 Scopus

TITLE-ABS-KEY ( ( "obstructive sleep apnea" OR "OSA" OR "sleep-disordered breathing" )

AND ( "total knee arthroplasty" OR "total hip arthroplasty" OR "TKA" OR "THA"

  OR "knee replacement" OR "hip replacement" OR "total joint arthroplasty" )

AND ( "complication" OR "pulmonary embolism" OR "deep vein thrombosis"

  OR "periprosthetic joint infection" OR "surgical site infection"

  OR "readmission" OR "revision" OR "length of stay" OR "CPAP" ) )

Database: Scopus. Last searched: May 1, 2026.

Total records retrieved: 400

A1.5 Database Overlap Assessment

Several included studies utilized large national administrative databases with potentially overlapping patient populations. The table below summarizes the database, study period, procedure, and OSA ascertainment method for each included study, along with an assessment of the risk of patient overlap with other included studies. No two included studies used the same database, procedure, time period, and OSA definition combination simultaneously. Three groups of studies share the same underlying database (PearlDiver Mariner) but differ in procedure (THA vs TKA vs TSA) and/or time period, making exact patient overlap unlikely but impossible to exclude entirely.

A prespecified sensitivity analysis excluding all CPAP-versus-non-OSA studies [6,11,13] - the subgroup most likely to share methodological similarities - was performed and is reported in the main text (Methods section). Results were not materially different for PE, DVT, or PJI outcomes, confirming robustness.

**Table 5:** Summary of the databases with risk assessment of patient overlap * Not pooled in primary TKA/THA meta-analyses. QS = qualitative synthesis only. SAF = Standard Analytical Files. PSM = propensity score matched. AHI = apnoea-hypopnoea index. CPAP = continuous positive airway pressure. Overlap risk classifications: Low = different database or clearly distinct cohort; Moderate = same database but different procedure/period; None = single institution. No studies were judged to have critical overlap risk that would preclude independent contribution to pooled estimates.

Study	Database	Study period	Procedure	OSA definition	N (OSA)	Overlap risk
Vakharia et al. (2019) [8]	Medicare SAF (via PearlDiver)	2005–2014	TKA & THA	ICD-9 code 327.23	189,968 TKA 74,652 THA	Low — Medicare SAF, separate from commercial PearlDiver Mariner
Mörwald et al. (2018) [17]	Premier Perspective DB	2006–2015	TKA & THA	ICD-9/ICD-10	107,610	Low — unique database not used by other studies
Sequeira et al. (2022) [6]	PearlDiver Mariner	2010–2020	THA	ICD-10 code G47.33	7,351 (CPAP) 7,351 (ctrl)	Moderate — shares PearlDiver Mariner with Bohn 2025 and McCormick 2023; THA-specific, different period
Tang et al. (2022) [7]	Single institution (NYU/RWJBarnabas)	2015–2019	TKA & THA	ICD-10/polysomnography	935	None — single institution
McCormick et al. (2023) [13]	PearlDiver Mariner	2010–2022	TKA	ICD-10 code G47.33	31,362 (CPAP) 31,362 (ctrl)	Moderate — shares PearlDiver Mariner with Sequeira 2022 and Bohn 2025; TKA-specific
Thomas et al. (2024)* [5]	SR/MA of 7 single-institution studies	2001–2022	TKA & THA	Varies by primary study	20,977 total	N/A — prior SR/MA; not pooled as primary study
Dubin et al. (2024) [11]	PearlDiver (commercial)	2015–2021	TKA	ICD-10 code G47.33 + CPAP code	Up to 169,225	Low — uses commercial PearlDiver (not Mariner); TKA-specific
Bohn et al. (2025) [9]	PearlDiver Mariner	2016–2022	THA	ICD-10 code G47.33	85,820	Moderate — shares PearlDiver Mariner with Sequeira 2022 and McCormick 2023; THA-specific, distinct period
Sterneder et al. (2025) [15]	Single institution (HSS, NY)	2017–2022	THA	Polysomnography AHI confirmed	1,141	None — single institution; QS only (no non-OSA comparator)
Fowler TKA (2025) [16]	TriNetX	2015–2023	TKA	ICD-10 code G47.33	27,976	Low — TriNetX is a distinct network; no overlap with PearlDiver studies
Fowler THA (2025) [10]	TriNetX	2015–2023	THA	ICD-10 code G47.33	19,501	Low — same TriNetX network as Fowler TKA; THA-specific arm; possible within-study overlap addressed by using separate TKA/THA estimates
Hand et al. (2025)* [14]	PearlDiver Mariner	2016–2022	TSA	ICD-10 code G47.33	28,559	N/A — shoulder arthroplasty; not pooled in TKA/THA analyses
Lu et al. (2025)* [12]	Single institution (China)	2019–2023	Spine (PLIF)	PSM + ICD-10	83	N/A — spine surgery; not pooled in TKA/THA analyses
Naqvi et al. (2017) [18]	Single institution (Rothman)	2007–2015	TKA & THA	ICD-9 code 327.23	1,246	None — single institution
Gupta et al. (2001) [19]	Single institution (Mayo Clinic)	1993–1998	TKA & THA	Polysomnography AHI ≥10	101	None — single institution
Lyons et al. (2016)* [20]	Single institution (Penn)	2013–2015	TKA & THA	Polysomnography confirmed	N/A (feasibility)	N/A — QS only; no poolable outcomes
Shen et al. (2021)* [21]	SR/MA of 6 RCTs	Variable	TKA & THA	N/A (sleep intervention, not OSA)	207 vs 209	N/A — not an OSA study; QS only

Supplementary Appendix 2: ROBINS-I risk of bias assessment

Risk of bias was assessed for all 17 included studies using the Risk of Bias in Non-Randomized Studies of Interventions (ROBINS-I) tool. Assessment was performed independently by two reviewers (NN, KS), with disagreements resolved by consensus (MB). ROBINS-I evaluates seven domains for each study; each domain is rated as Low (L), Moderate (M), Serious (S), or Critical (C) risk of bias. Studies classified as qualitative synthesis only [QS] and contextual studies are included for transparency; their domain ratings are provided, but they did not contribute to pooled estimates. Thomas et al. [5] is a prior systematic review and meta-analysis, not a primary study; it is assessed for reference only.

Domain definitions (ROBINS-I):

• D1 - Confounding: Potential for pre-existing differences between OSA and non-OSA groups not accounted for by the study design. The main confounders of concern are BMI/obesity, diabetes mellitus, cardiovascular disease, age, and frailty.

• D2 - Selection of participants: Whether the selection of study participants into the study could be related to both the exposure (OSA) and the outcome. Main concern: detection bias - OSA is more likely to be diagnosed in patients who are more symptomatic or higher-risk.

• D3 - Classification of interventions: Whether OSA was correctly classified. Main concerns: ICD code-based ascertainment misses undiagnosed OSA; AHI severity not captured; CPAP prescription used as OSA surrogate in some studies.

• D4 - Deviations from intended interventions: For OSA studies, the 'intervention' is OSA status, not a modifiable intervention; rated NA for all observational studies not evaluating CPAP effects. Rated M for CPAP subgroup studies where cross-over or non-adherence is possible.

• D5 - Missing data: Completeness of outcome data. Administrative database studies typically have near-complete follow-up within the database period; rated Low for most. Single-institution cohort studies may have higher attrition.

• D6 - Measurement of outcomes: Whether outcomes were measured consistently between OSA and non-OSA groups. ICD-code-based outcome ascertainment used in all database studies; comparable coding practices assumed between groups.

• D7 - Selection of reported results: Whether results were selectively reported. Rated Moderate for most studies as pre-registration was not universal; no evidence of selective outcome suppression identified in any study.

**Table 6:** ROBINS-I domain ratings of the included studies L = Low risk; M = Moderate risk; S = Serious risk; C = Critical risk; NA = Not applicable D1: Confounding | D2: Selection of participants | D3: Classification of interventions | D4: Deviations from intended interventions | D5: Missing data | D6: Measurement of outcomes | D7: Selection of reported results. (QS) = qualitative synthesis only (not pooled). (contextual) = included for narrative synthesis only; not in primary TKA/THA pool. (SR/MA) = prior systematic review and meta-analysis; assessed for reference; not pooled as primary study. Confounding (D1) was rated 'Serious' for database studies using OSA ICD codes without propensity matching (Sequeira et al. [6], McCormick et al. [13], Dubin et al. [11], Mörwald et al. [17], Gupta et al. [19]) because key confounders, including BMI, diabetes, cardiovascular disease, and frailty, may not have been adequately controlled. Studies using propensity score matching on multiple covariates (Bohn et al. [9], Fowler et al. [10,16], Vakharia et al. [8], Naqvi et al. [18], Tang et al. [7]) were rated Moderate as residual confounding persists. Classification of interventions (D3) was rated 'Moderate' for all ICD-code-based studies because up to 80% of OSA patients in the general population remain undiagnosed, creating systematic misclassification of the unexposed group.

Study	D1 Conf.	D2 Select.	D3 Class.	D4 Deviations	D5 Missing	D6 Outcome Measure	D7 Reporting	Overall
Gupta et al. (2001) [19]	S	M	M	NA	M	M	M	S
Naqvi et al. (2017) [18]	M	M	M	NA	M	M	M	M
Mörwald et al. (2018) (QS) [17]	S	M	M	NA	M	M	M	S
Lyons et al. (2016) (QS) [20]	M	M	NA	NA	L	M	M	M
Vakharia et al. (2019) [8]	M	M	M	NA	L	M	M	M
Shen et al. (2021) (QS) [21]	L	L	L	M	L	M	L	M
Sequeira et al. (2022) [6]	S	M	M	NA	L	M	M	S
Tang et al. (2022) [7]	M	M	M	NA	M	M	M	M
McCormick et al. (2023) [13]	S	M	M	NA	L	M	M	S
Thomas et al. (2024) (SR/MA) [5]	M	L	L	NA	M	M	M	M
Dubin et al. (2024) [11]	S	M	M	NA	L	M	M	S
Bohn et al. (2025) [9]	M	M	M	NA	L	M	M	M
Sterneder et al. (2025) (QS) [15]	L	M	L	NA	L	M	M	M
Fowler et al. TKA (2025) [16]	M	M	M	NA	L	M	M	M
Fowler et al. THA (2025) [10]	M	M	M	NA	L	M	M	M
Lu et al. (2025) (contextual) [12]	M	M	M	NA	M	M	M	M
Hand et al. (2025) (contextual) [14]	M	M	M	NA	L	M	M	M

A2.2 Summary of Risk of Bias by Outcome Pool

Outcome

Overall ROBINS-I (pool)

Dominant bias concern

Pulmonary embolism (k=7)

Moderate

Confounding by BMI and cardiovascular disease (D1); PSM-adjusted studies partially mitigate. Classification bias from ICD-coding of OSA (D3).

Deep vein thrombosis (k=7)

Moderate-high

Same as PE; additionally high heterogeneity across comparator groups (OSA vs non-OSA; CPAP vs non-OSA) contributes to ROBINS-I uncertainty.

PJI (k=4)

Moderate

Confounding by diabetes, immunosuppression, and prior infection (D1); partially addressed by matching in Bohn 2025 and Vakharia 2019.

SSI (k=2, narrative)

Moderate

Limited to two studies; broader inference not possible. Lu 2025 (spine) excluded from pool.

90-day readmission (k=7)

High

Comparator heterogeneity (three distinct comparators); Sequeira 2022 rated Serious for D1 (disease-severity selection). I²=100%.

Revision arthroplasty (k=4)

Moderate-high

Follow-up heterogeneity (1-2 years); revision ascertainment via ICD codes may be incomplete.

Length of stay (k=11)

Moderate

Consistent direction across studies; LOS influenced by unmeasured factors, including monitoring protocols and institutional policies.

ROBINS-I overall judgment for the pool is based on the highest risk study contributing to that pool and the nature of the dominant bias concern. Moderate = results likely represent true associations, but residual confounding cannot be excluded. Moderate-high = results probably valid, but comparator heterogeneity or classification bias materially affects precision. High = pooled OR should be interpreted with caution; the I²=100% readmission estimate is particularly sensitive to Sequeira et al. [6].
